# MRI quality control for low‐field MR‐IGRT systems: Lessons learned

**DOI:** 10.1002/acm2.12713

**Published:** 2019-09-21

**Authors:** H. Michael Gach, Austen N. Curcuru, Erin J. Wittland, Borna Maraghechi, Bin Cai, Sasa Mutic, Olga L. Green

**Affiliations:** ^1^ Department of Radiation Oncology Washington University in St. Louis St. Louis Missouri 63110 USA; ^2^ Department of Radiology Washington University in St. Louis St. Louis Missouri 63110 USA; ^3^ Department of Biomedical Engineering Washington University in St. Louis St. Louis Missouri 63110 USA; ^4^ Department of Radiation Oncology Barnes Jewish Hospital St. Louis Missouri 63110 USA

**Keywords:** low‐field, MRI, MR‐IGRT, quality control

## Abstract

**Purpose:**

To present lessons learned from magnetic resonance imaging (MRI) quality control (QC) tests for low‐field MRI‐guided radiation therapy (MR‐IGRT) systems.

**Methods:**

MRI QC programs were established for low‐field MRI‐^60^Co and MRI‐Linac systems. A retrospective analysis of MRI subsystem performance covered system commissioning, operations, maintenance, and quality control. Performance issues were classified into three groups: (a) Image noise and artifact; (b) Magnetic field homogeneity and linearity; and (c) System reliability and stability.

**Results:**

Image noise and artifacts were attributed to room noise sources, unsatisfactory system cabling, and broken RF receiver coils. Gantry angle‐dependent magnetic field inhomogeneities were more prominent on the MRI‐Linac due to the high volume of steel shielding in the gantry. B_0_ inhomogeneities measured in a 24‐cm spherical phantom were <5 ppm for both MR‐IGRT systems after using MRI gradient offset (MRI‐GO) compensation on the MRI‐Linac. However, significant signal dephasing occurred on the MRI‐Linac while the gantry was rotating. Spatial integrity measurements were sensitive to gradient calibration and vulnerable to shimming. The most common causes of MR‐IGRT system interruptions were software disconnects between the MRI and radiation therapy delivery subsystems caused by patient table, gantry, and multi‐leaf collimator (MLC) faults. The standard deviation (SD) of the receiver coil signal‐to‐noise ratio was 1.83 for the MRI‐^60^Co and 1.53 for the MRI‐Linac. The SD of the deviation from the mean for the Larmor frequency was 1.41 ppm for the MRI‐^60^Co and 1.54 ppm for the MRI‐Linac. The SD of the deviation from the mean for the transmitter reference amplitude was 0.90% for the MRI‐^60^Co and 1.68% for the MRI‐Linac. High SDs in image stability data corresponded to reports of spike noise.

**Conclusions:**

There are significant technological challenges associated with implementing and maintaining MR‐IGRT systems. Most of the performance issues were identified and resolved during commissioning.

## 1 INTRODUCTION

In 2014, the first patient was treated with ViewRay’s MRIdian integrated ^60^Co 0.35 T magnetic resonance imaging (MRI) guided radiotherapy (MR‐IGRT) system.^1^ Since 2017, commercial MRI linear accelerators (MRI‐Linacs) with magnetic fields of 0.35 T (ViewRay MRIdian) and 1.5 T (Elekta Unity) have been treating patients.^2^, ^3^

Quality assurance (QA) and quality control (QC) guidelines for MRI are addressed by the American College of Radiology (ACR),^4^ the American Association of Physicists in Medicine (AAPM),^5^ and the National Electrical Manufacturers Association (NEMA) standards.^6^ Separate QA guidelines are available for conventional Linacs.^7^ AAPM Task Group 117 is tasked with developing MRI QC guidelines for treatment planning and stereotactic radiation therapy (RT). QC results for MR‐IGRT were reported for the ViewRay 0.35 T MRI‐^60^Co [Ref. 8] and MRI‐Linac,^3^ and the 1.5 T Elekta Unity.^9^

The quality of the MRI was previously reported to be satisfactory for both commercial low‐field MR‐IGRT systems.^8^, ^10^ However, a lot of time and work was required during the implementation and commissioning of the MRI‐^60^Co and MRI‐Linac systems to resolve performance issues prior to clinical operations. In the process, much was learned about system deficiencies and fixes that benefitted manufacturing, installation, QC procedures, and future system development.

The purpose of this study is to present the lessons learned from commissioning, operating, and performing quality control on 0.35 T MRI‐^60^Co and MRI‐Linac MR‐IGRT systems. These lessons will be categorized herein as: (a) Image noise and artifact associated with electromagnetic interference (EMI) sources; (b) Field homogeneity and linearity and their effects on image spatial integrity; and (c) System reliability and stability issues.

## 2 MATERIAL AND METHODS

Data were acquired on ViewRay MRI‐^60^Co (13.6 MHz) and 6 MV MRI‐Linac (14.7 MHz) systems (Oakwood Village, OH). The MRI‐^60^Co has three depleted uranium‐encased ^60^Co heads positioned 120^0^ apart around the gantry.^11^ The MRI‐Linac has six 227‐kg steel shields positioned 60^0^ apart around the gantry.^3^ Both models are shimmed to ≤25 ppm pk‐pk over a 45‐cm diameter spherical volume (DSV) at each gantry angle using five higher‐order superconducting shims and passive shim trays located in the gradient assembly. The MRI‐Linac also uses passive shims oriented around, and mounted to, the rotating gantry to shim the steel shields. Gradient shimming is used to reduce the field inhomogeneity to <5 ppm in a 24 cm diameter spherical phantom.

Three commercial water phantoms were used in these studies: a) Large ACR phantom (J.M. Specialty Parts, San Diego, CA);^4^ b) Fluke 76‐907 uniformity linearity water phantom doped with 15 mM CuSO_4_ (HP Manufacturing, Cleveland, OH); and c) Siemens 24‐cm diameter spherical water phantom doped with 5 mM NiSO_4_.

### 2.1 Image noise and artifact

During the commissioning of the MRI‐Linac, we investigated sources of EMI using the three commercial phantoms. The effects of EMI from B_0_ instabilities on signal averaging were investigated for the MRI‐Linac using the large ACR phantom and *in vivo* with the torso phased array receiver coils (with body coil transmission).

Both MR‐IGRT models currently average two images to produce a 2D cine frame. The reasons for averaging are twofold: a) The original image processing (target tracking and beam gating) pipeline could not handle a throughput >4 frames per second (fps); and b) The averaged images provide enhanced signal‐to‐noise ratio (SNR) vs single acquisitions. Long‐term averaging acquires the k‐space from one image followed by the k‐space from the second image, then combines the two k‐space datasets and reconstructs the averaged image. Short‐term averaging acquires a line of k‐space for the first image followed by the same line of k‐space for the second image, and then increments the phase‐encode line to acquire the full k‐space in this manner. Averaging can cause or mitigate image artifacts depending on the source of the variation (e.g., physiological motion) and the type of averaging.^12^

### 2.2 Field homogeneity and linearity

The MRI‐^60^Co employs a tune‐up shim mode that uses phantom‐based field homogeneity measurements for patient shimming for both 2D and 3D acquisitions. The gradient offsets (first‐order shim terms) do not vary with gantry angle.

The MRI‐Linac shimming represents two changes from the MRI‐^60^Co. First, a standard shim is performed for each patient prior to each 3D acquisition used in treatment planning and setup. The standard shim mode acquires a field map in the patient and calculates the first‐order shim currents that will provide the optimal field homogeneity for the imaging volume. Second, a phantom‐based shim adjustment that varies with gantry angle is used for the 2D cine treatment acquisitions and is named MRI gradient offset (MRI‐GO). In MRI‐GO, the first‐order shim currents are updated as the gantry position changes based on a lookup table of gantry angles and corresponding first‐order shim current settings calculated using the 24‐cm diameter spherical phantom.

Field homogeneity was measured for gantry angles varying from 0 to 150^0^ on the MRI‐^60^Co and 0‐345^0^ on the MRI‐Linac in 15^0^ increments using the spherical phantom. Measurements were made using both the tune‐up and standard shim modes. The corresponding first‐order shim values were also recorded. A free induction decay (FID) was acquired with the sphere centered at isocenter (TE/TR: 0.35 ms/3 s, Flip angle: 90^0^, 4 Averages, 5 Hz/point, 256 complex points). The proton spectra were fit to a Lorentzian function using a nonlinear fit algorithm, and the full width at half maximum fits were then calculated.

The original magnetic field homogeneity specification for the Functional Test Procedure (FTP) was baseline +/−1.5 ppm for the MRI‐^60^Co and ≤5 ppm for the MRI‐Linac using the tune‐up shim mode. In general, the current field homogeneity target is ≤2 ppm for all gantry angles.

Spatial integrity measurements were made using the manufacturer‐provided uniformity linearity phantom and the body coil for image transmission and reception. The phantom was available in two formats: one with square holes and one with round holes. Both were used herein.

The spatial integrity tests were performed by centering the grid portion of the uniformity linearity phantom at seven positions relative to isocenter (axial orientation with z = 0, coronal orientation with y = 0, and sagittal orientations at x = −12.5, −7, 0, 7, and 12.5 cm). A proprietary software program (ViewRay, Oakwood Village, OH) was used to analyze the uniformity linearity phantom for compliance (within +/−1 mm error for ≤10 cm DSV and within +/−2 mm for diameters between 10 and 20 cm DSV). Measurements were also conducted for varying gantry angles in increments of 30^0^ to assess the stability of the spatial integrity.

### 2.3 System reliability and stability

Common reliability issues were documented from maintenance logs of MRI subsystem failures. A large homebuilt phantom (Fig. 15) was used to test the individual phased array coil elements every month and when a coil was suspected to be malfunctioning. Sixteen 6‐cm diameter holes, forming a 4x4 grid, were cut into a 61 cm × 61 cm × 13 cm polyurethane foam block (Grainger). A phantom bottle filled with water doped with NiCl_2_ (Philips Healthcare, Part # 45980006937x) was inserted into each hole. The phantom was placed on the patient table with the coil under test (CUT) placed on top. The uniformity linearity phantom was placed on top of the coil for loading and to provide a source of proton signal for the MRI prescan calibrations.

System stability was assessed based on monthly measurements of the Larmor frequency, RF reference amplitude, SNR of the torso coils, and image stability. The SNR was calculated using the two‐image difference method and a region of interest (ROI) that covered 75% of the area in a homogeneous slice of the large ACR phantom (Slice 7 of 11 from the ACR QC prescription).^4^ The SNR was calculated using the mean signal in the ROI (*<Signal>*) from the first image and the standard deviation (*SD*) of the noise in the difference image (*σ_Noise_*):

(1) $$SNR=\frac{\sqrt{2}\cdot \left\langle Signal\right\rangle }{\sigma_{\mathrm{Noise}}}$$

The stability in the Larmor frequency and reference amplitude (as a surrogate for transmitter gain) was measured as the deviation from the mean to compensate for changes resulting from reramping the MRI‐^60^Co magnet. The Larmor frequency and reference amplitude were obtained from the monthly QC measurements of the large ACR phantom.

An image stability scan was run monthly using the large ACR phantom to identify RF spikes or other instabilities while the MRI was stressed.^13^ A sagittal 2D cine TrueFISP sequence was run (TE/TR: 1/2 ms, flip angle: 60^0^, 1335 Hz/pixel, 3.5 × 3.5 × 7 mm, GRAPPA 2, 3 slices, 0.125 s/image, no averaging, 300 repetitions, 123 s). The mean signal in a 5‐cm diameter ROI in the center of the middle slice was calculated. The standard deviation was calculated from the mean values across the repetitions as a metric for image stability.

## 3 RESULTS

### 3.1 Image noise and artifact

**Figure 1:**
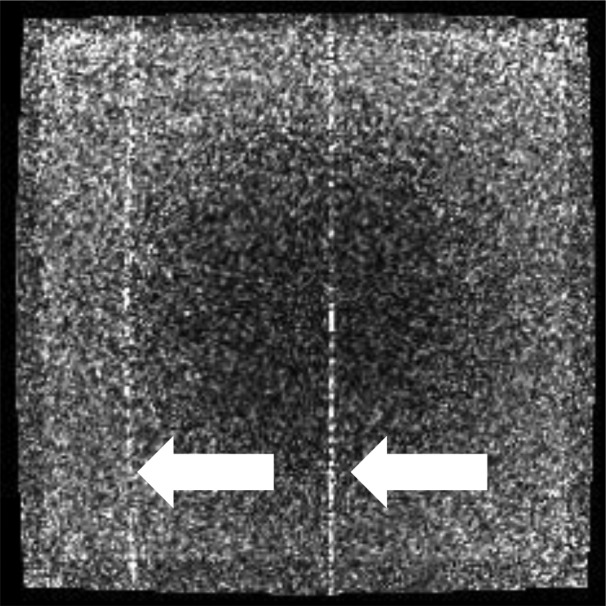
Axial 3D TrueFISP (TE/TR: 1/3 ms, 60^0^, 1.5 × 1.5 × 3 mm, 534 Hz/pixel, 81 s) using the body coil for reception. RF noise appears as line (zipper) artifacts that run along the phase‐encode direction thus indicating it is caused by a continuous RF source. The image plane was 7 mm outside of the uniformity linearity phantom. The noise was caused by Model LTC‐8640‐10M data filters (ETS Lindgren, Cedar Park, TX) installed in the penetration panel with passbands (0–25 MHz) that included the MRI Larmor frequency. MRI, magnetic resonance imaging.

**Figure 2:**
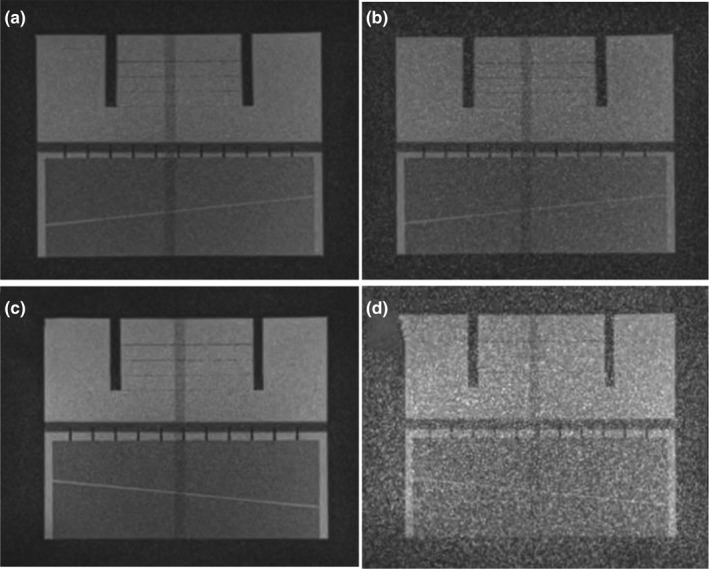
Effect of patient table noise on MRI‐^60^Co (a, b) and MRI‐Linac (c, d) using the large ACR phantom. The patient table was turned off (a, c) and then powered on (b, d). Coronal images from three‐plane T_1_‐weighted localizers are shown (TE/TR: 20/200 ms, 1 × 1 × 5 mm, flip angle: 90^0^, 78 Hz/pixel, 53 s). The window levels and widths are identical for all of the images. ACR; American College of Radiology; MRI, magnetic resonance imaging.

**Figure 3:**
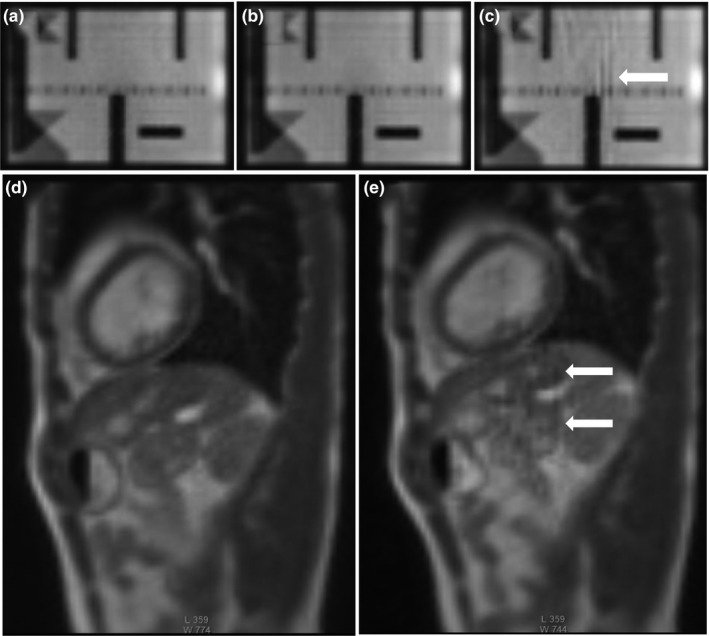
Sagittal 2D TrueFISP cine acquisition (TE/TR: 1/2 ms, flip angle: 60^0^, 1335 Hz/pixel, 3.5 × 3.5 × 7 mm, 2 averages, GRAPPA 2) from the MRI‐Linac acquired at gantry angle 240^0^ in the ACR phantom (a–c) and a 34‐year‐old male volunteer (d, e) using MRI‐GO. (a) no averaging, 125 ms/frame. (b, c) two averages, 250 ms/frame with (b, d) short‐term averaging and (c, e) long‐term averaging with streaking artifacts. The severity of the streaking artifacts depends on the gantry angle. The window levels and widths are identical for all of the corresponding images. ACR; American College of Radiology; MRI‐GO; MRI gradient offset.

**Figure 4:**
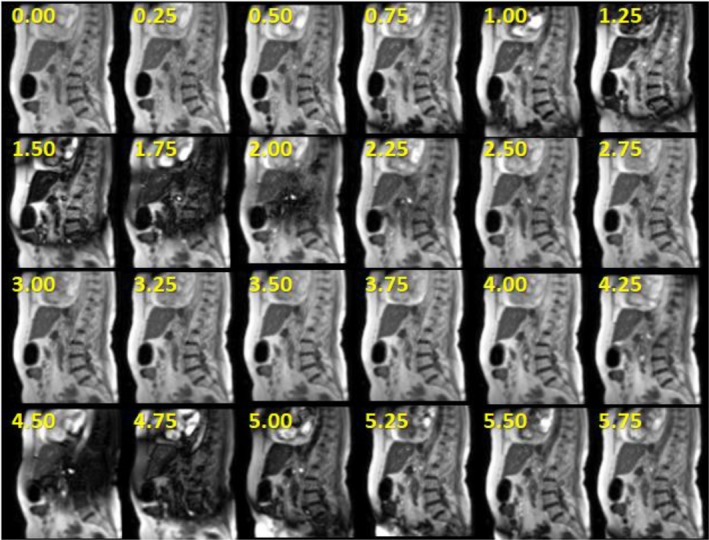
EMI‐related (moving metal) dephasing artifacts that occurred during gantry angle rotation from 300^0^ to 320^0^ in 76‐year‐old female patient receiving adaptive MR‐IGRT for pancreatic cancer on the MRI‐Linac. The numbers represent the time in seconds corresponding to each frame during the gantry rotation. The cine images used 2D sagittal TrueFISP cine (TE/TR: 0.91/2.10 ms, 60^0^, GRAPPA 2, 3.5 × 3.5 × 7 mm, 1351 Hz/pixel, 2 averages, 4 frames/s). Radiation delivery is paused during gantry rotation. Therefore, there is no degradation in treatment accuracy.

Sources of EMI that affected MRI quality are summarized in Table 1. Examples of EMI in MRI are shown in Figs. 1, 2. A comparison of short‐term and long‐term averaging for the MRI‐Linac is shown in Fig. 3. Depending on the gantry angle, streaking artifacts were observed in MRIs acquired using long‐term averaging regardless of the shim mode. The effects of quasi‐static EMI associated with gantry rotation in the MRI‐Linac are shown in Fig. 4. The dephasing artifacts tend to occur in pairs during gantry rotation.

**Table 1:** Historical sources of EMI by 0.35 T MR‐IGRT model.

Source	Mechanism	Manifestation	^60^Co	Linac
Cables	Poor or broken shielding	Image noise and artifact	✓	✓
Dose monitor ionization chamber signal amplifier	Poorly shielded power cable	Image noise		✓
Gantry steel	B_0_ instabilities	Image (dephasing) artifacts during gantry rotation		✓
Gradient thermal sensors	Gradient and B_0_ instabilities	Image artifacts		✓
RF Coils	Broken components	Signal loss. Image noise and artifacts.	✓	✓
Patient table	Poor shielding	Image noise	✓	✓
Multi‐leaf collimators (MLC)	Motor and power noise	Image artifacts during MLC motion	✓	✓
Signal filters	Improper specification	Image noise and artifacts		✓
Magnetron tuning rod motor	Pulsing during MRI	Image noise and artifacts		✓
RF waveguide	Missing RF gasket	Image noise and artifacts		✓

Abbreviations: EMI, electromagnetic interference; MR‐IGRT; MRI‐guided radiation therapy.

### 3.2 Field homogeneity and linearity

**Figure 5:**
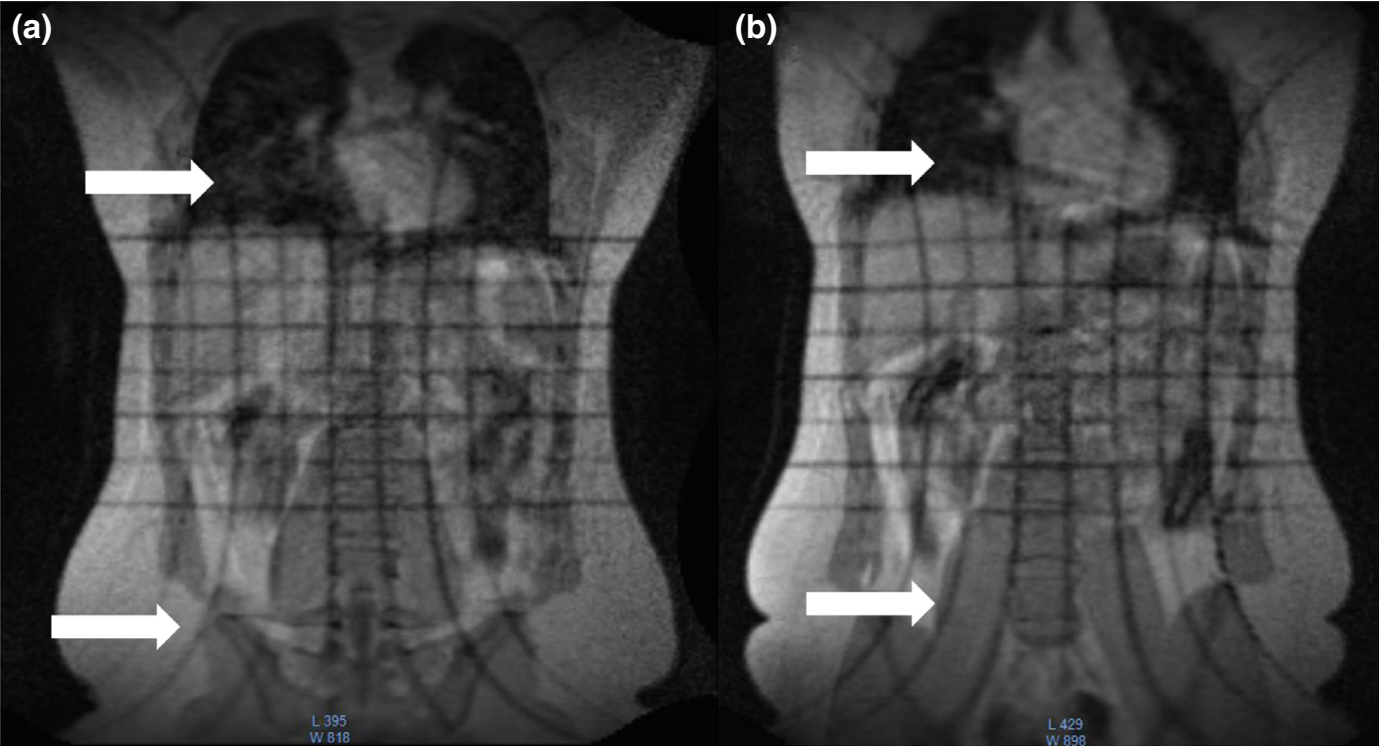
2D T_1_‐weighted gradient echo coronal MRI localizer (TE/TR: 3.44/147 ms, 2 × 2 × 5 mm, gap: 25 mm, flip angle: 60^0^, 300 Hz/pixel, 7 slices/orientation, 28 s) showing axial and sagittal (arrows) slice excitation profiles *in vivo*. Gradient nonlinearities cause the slice profiles to curve away from isocenter at large offsets from isocenter. The nonlinearities are comparable between the MRI‐^60^Co (a) and the MRI‐Linac (b). The gradient nonlinearities emphasize the importance of placing the target as close to isocenter as practical to minimize their effect on geometric accuracy. MRI, magnetic resonance imaging.

**Figure 6:**
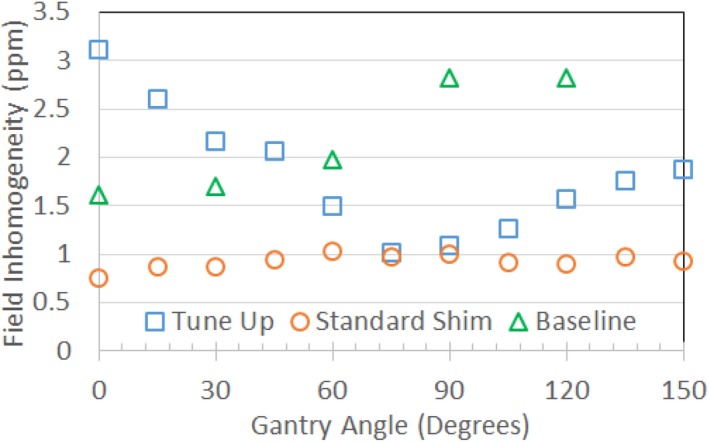
Comparison of field inhomogeneities (spectral FWHM) vs gantry angle for the MRI‐^60^Co measured with the 24‐cm sphere. Tune‐up shim values (squares) and the November 2013 baseline measurements (triangles) used fixed shim settings for all gantry angles based on a phantom calibration. Measurements were also made using the standard shim mode (circles) for comparisons even though the mode is not used for MRI‐^60^Co therapy. The tune‐up and standard shim measurements were conducted in March 2019. MRI, magnetic resonance imaging.

**Figure 7:**
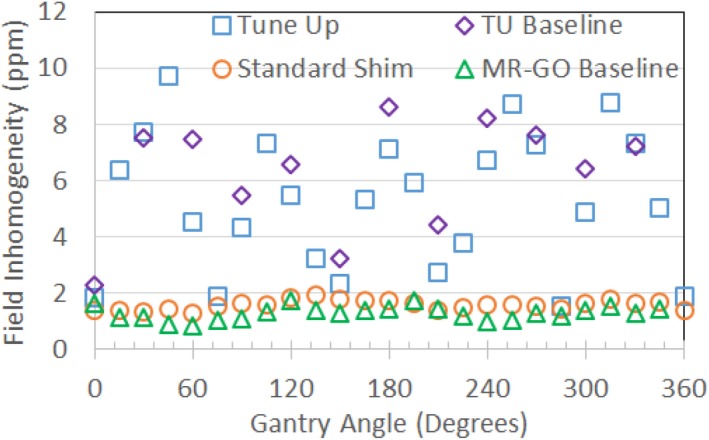
Comparison of field inhomogeneities (spectral FWHM) vs gantry angle for the MRI‐Linac measured with the 24‐cm sphere in March 2019. Tune‐up (TU) shim values (squares) used a fixed phantom calibration for all gantry angles. The standard shim mode (circles) reshimmed the field at each gantry angle using the gradients. The MRI‐GO baseline measurements (triangles) were acquired using the standard shim mode in January 2018. The TU baseline (diamonds) was measured in February 2018 using the tune‐up shim values. MRI, magnetic resonance imaging.

Figure 5 illustrates the effects of gradient nonlinearities on 2D slice excitations. The tune‐up and standard shim field inhomogeneity measurements for different gantry angles are shown in Fig. 6 for the MRI‐^60^Co and Fig. 7 for the MRI‐Linac. Technically, the MRI‐^60^Co and the MRI‐Linac tune‐up shim values do not comply with the specification of baseline +/−1.5 ppm. The MRI‐^60^Co inhomogeneities using the tune‐up shim mode fall within 5 ppm while the MRI‐Linac does not. Standard shims at each gantry angle permit the field inhomogeneity to be ≤2 ppm for both systems.

**Figure 8:**
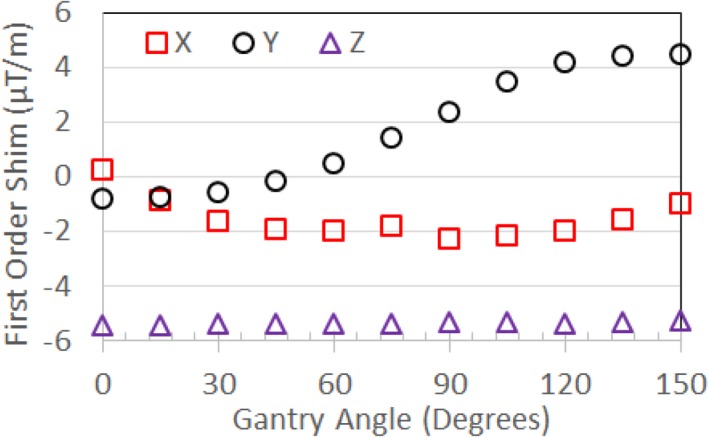
First‐order term (gradient) shim settings for standard shim measurements for the MRI‐^60^Co. A significant variation in Y shimming with gantry angle is observed. MRI, magnetic resonance imaging.

**Figure 9:**
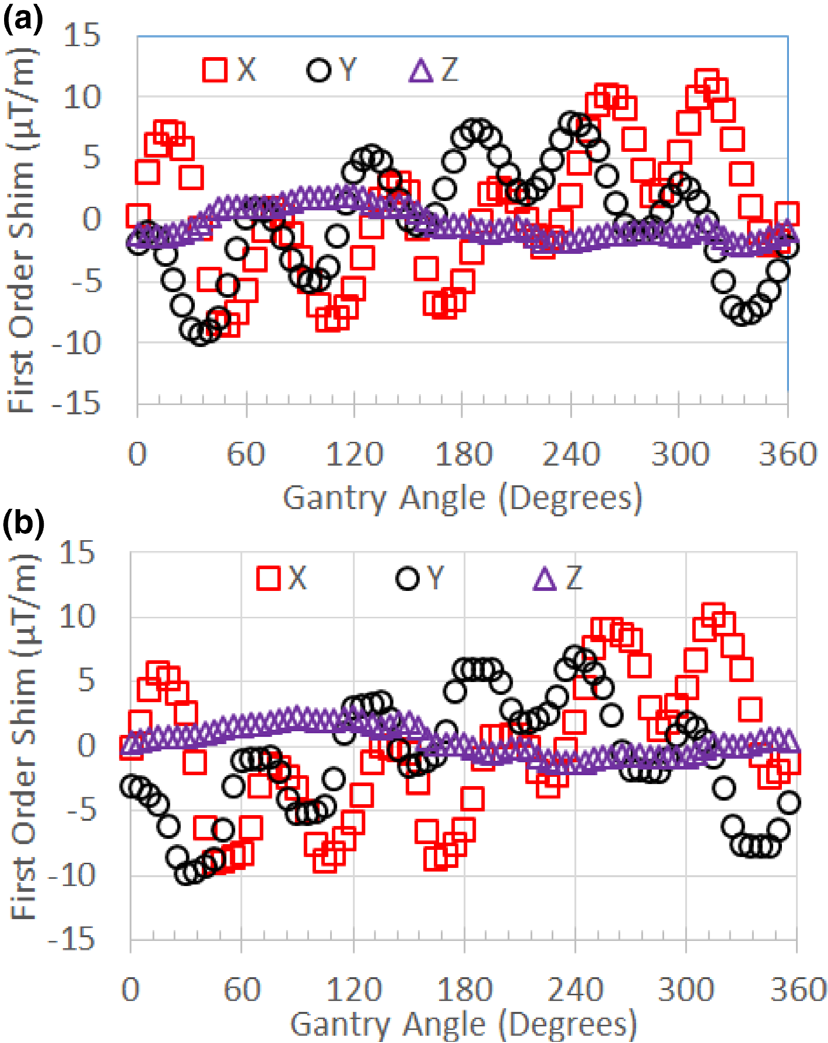
(a) First‐order term (gradient) shim settings for standard shim measurements for the MRI‐Linac (March 2019) interpolated to 5^0^ increments in gantry angle. (b) First‐order term shim settings for MRI‐GO (January 2018). Variations related to the 60^0^ spacing of the steel shields are evident. MRI, magnetic resonance imaging; MRI‐GO; MRI gradient offset.

The first‐order standard shim values are shown in Fig. 8 for the MRI‐^60^Co for various gantry angles. Figure 9 compares the March 2019 first‐order standard shim values for the MRI‐Linac interpolated to 5^0^ gantry angle increments using cubic interpolation to the January 2018 MRI‐GO first‐order standard shim values.

**Figure 10:**
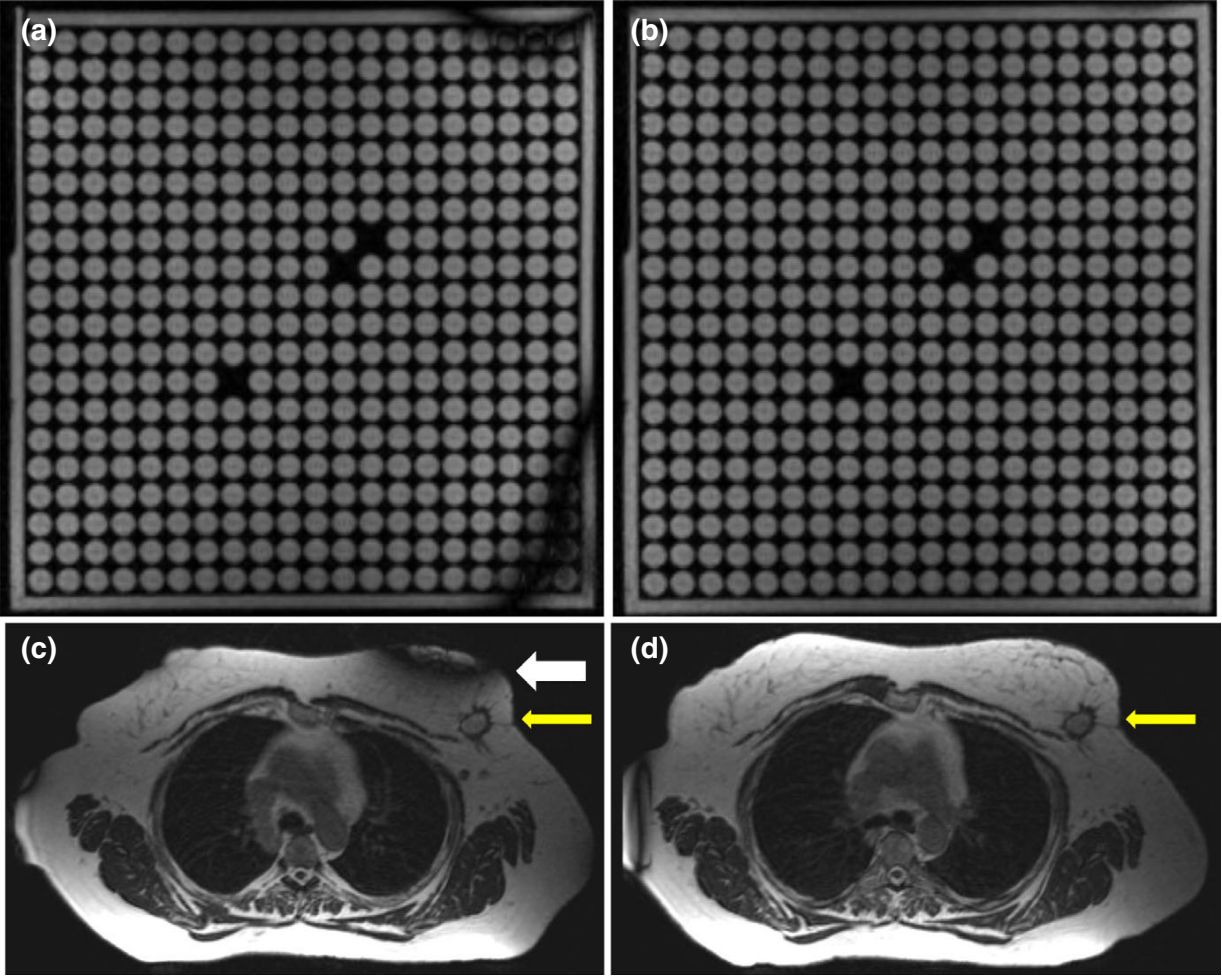
Comparison of (a) tune‐up and (b) standard shim modes on image quality in the uniformity linearity phantom on the MRI‐Linac. Images were acquired using axial 3D TrueFISP (TE/TR: 1/3 ms, 60^0^, 1.5 × 1.5 × 3 mm, 534 Hz/pixel, 81 s) with the body coil for reception. In this case, the tune‐up shim field inhomogeneities caused null bands. (c) Example of null band artifacts (large arrow) in the treatment field of view (c) of a 62‐year‐old patient with a breast malignancy (indicated by thin arrow) imaged on the MRI‐Linac at gantry angle 27^0^. The artifact was eliminated by changing the gantry angle to 0^0^ (d). Images (c, d) were acquired using axial 3D TrueFISP (TE/TR: 1/3 ms, 60^0^, 1.5 × 1.5 × 3 mm, 535 Hz/pixel, 274 slices, 172 s) with the standard shim mode. The window levels and widths are identical for all of the corresponding images. MRI, magnetic resonance imaging.

**Figure 11:**
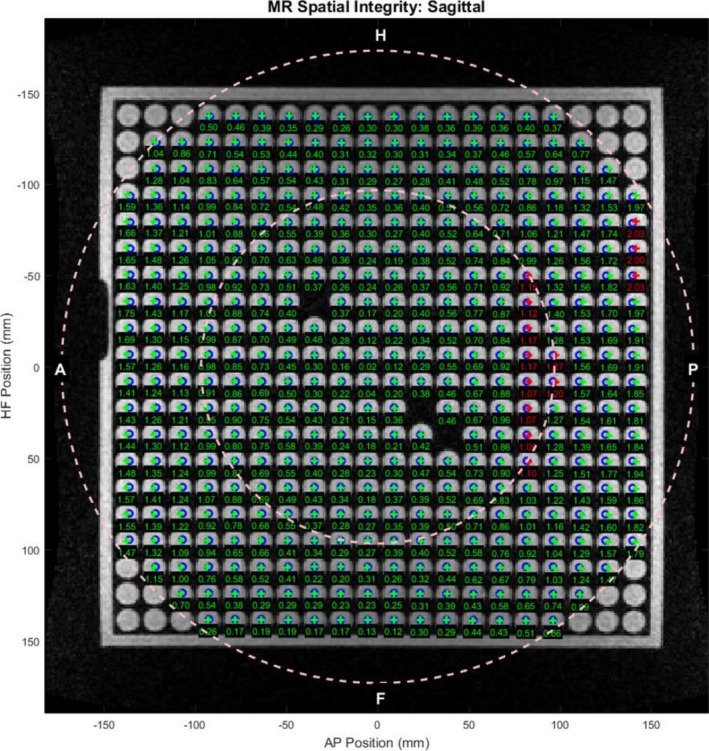
Software analysis results for sagittal 3D TrueFISP slice at x = 0 (TE/TR: 1/3 ms, 60^0^, 1.5 × 1.5 × 3 mm, 534 Hz/pixel, 81 s) on the MRI‐Linac using standard shimming at gantry angle 0^0^. Green indicates a passing score for the location while red is failing. The failed test was caused by improper calibration of the Y‐gradient. MRI, magnetic resonance imaging.

**Figure 12:**
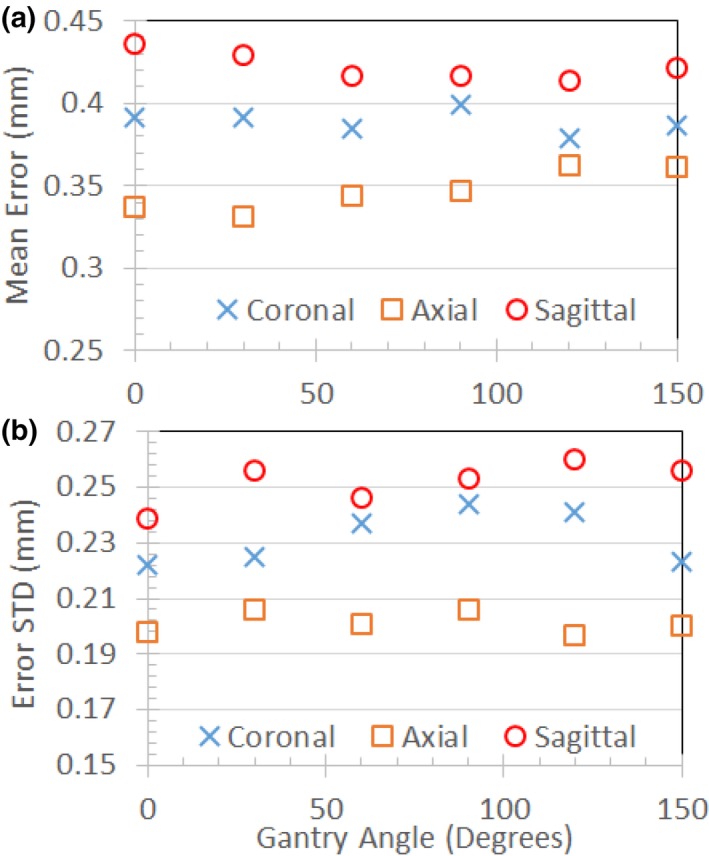
Mean (a) and standard deviation (b) for geometric errors measured in the intersection of a 40 cm DSV with the uniformity linearity phantom on the MRI‐^60^Co using tune‐up shim mode. Measurements were made with the phantom's grid positioned at isocenter in coronal, axial, and sagittal orientations. DSV, diameter spherical volume; MRI, magnetic resonance imaging.

**Figure 13:**
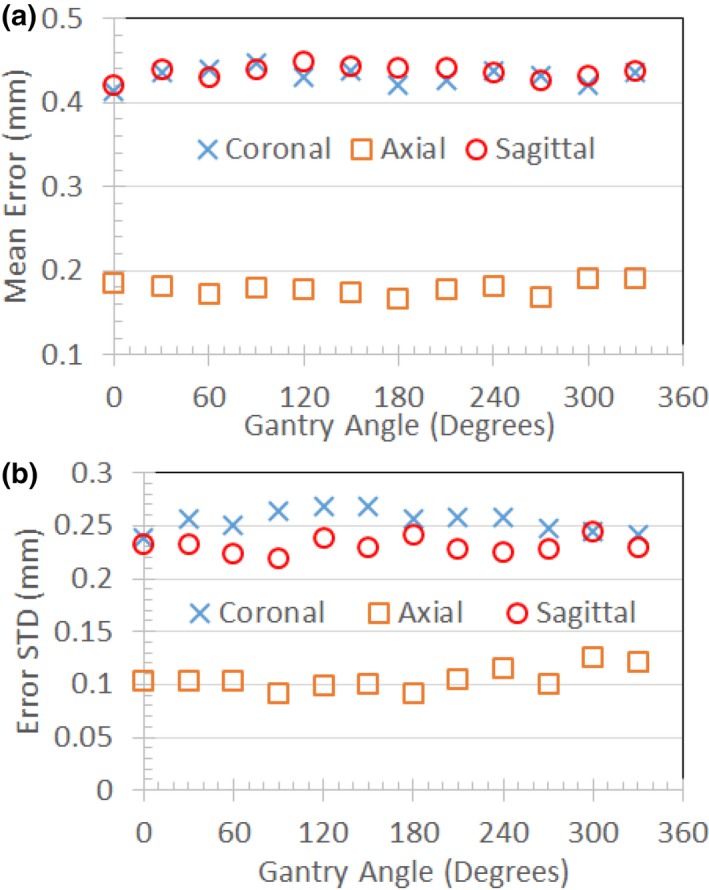
Mean (a) and standard deviation (b) for geometric errors measured in the intersection of a 40 cm DSV with the uniformity linearity phantom on the MRI‐Linac using standard shim mode. Measurements were made with the phantom's grid positioned at isocenter in coronal, axial, and sagittal orientations. DSV, diameter spherical volume; MRI, magnetic resonance imaging.

Figure 10(a) and 10(b) compares the effects of tune‐up vs standard shim modes on image quality in the uniformity linearity phantom for the MRI‐Linac. Figure 10(c) and 10(d) presents the effect of gantry angle on artifact in a breast cancer patient while using the standard shim mode. Figure 11 shows an example of spatial integrity not meeting the specification due to improper gradient calibration. Figures 12, 13 show the dependence of spatial integrity errors on gantry angle for the MRI‐^60^Co and MRI‐Linac, respectively.

### 3.3 System reliability and stability

**Figure 14:**
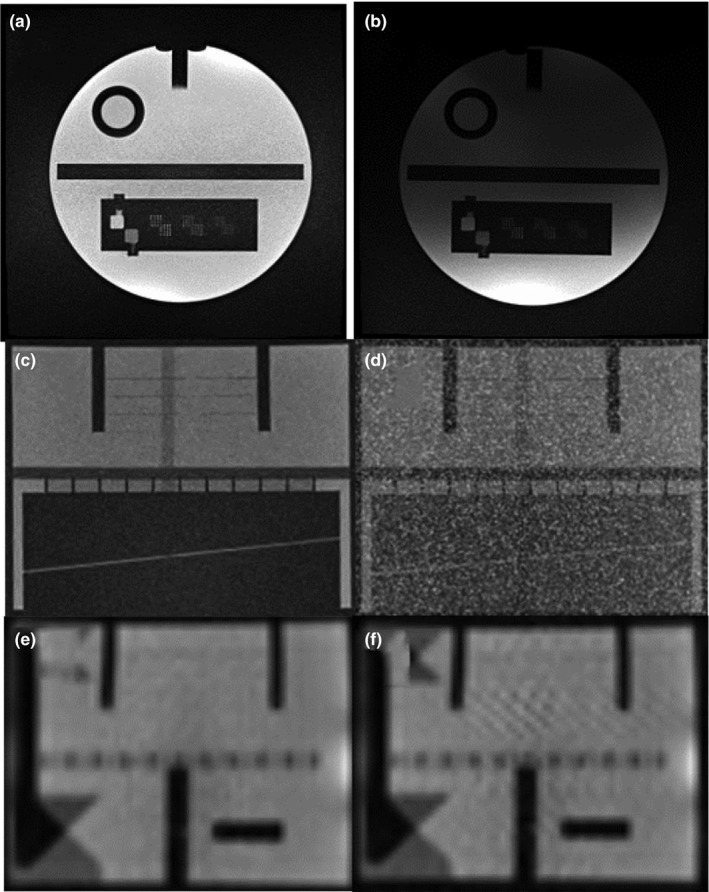
Comparison of MRIs acquired on MRI‐^60^Co with operational (a, c, e) and defective (b, d, f) torso array coils. (a, b) Axial T_1_‐weighted MRIs (TE/TR: 20/500 ms, 90^0^, 1 × 1 × 5 mm, 78 Hz/pixel, 260 s) with (b) defective anterior torso coil with severe signal loss. (c, d) Coronal T_1_‐weighted MRIs (TE/TR: 20/200 ms, 90^0^, 1 × 1 × 5 mm, 78 Hz/pixel, 53 s) with (d) excessive noise. (e, f) 2D sagittal TrueFISP (TE/TR: 1/2 ms, 60^0^, 3.5 × 3.5 × 7 mm, 1335 Hz/pixel, GRAPPA 2, 0.25 s) with (f) herringbone artifact (near center) indicative of RF spike noise. MRI, magnetic resonance imaging.

**Figure 15:**
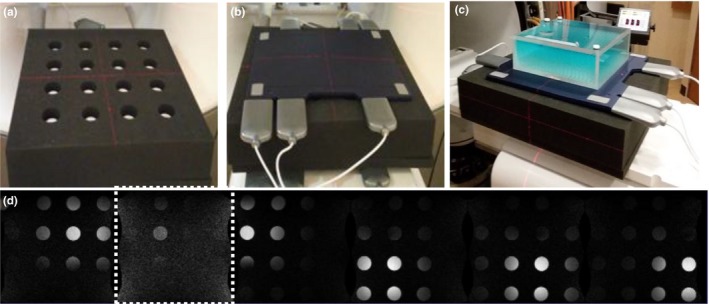
Phased array coil QC. (a) The 61 × 61 × 13 cm polyurethane foam phantom is shown with the 6‐cm diameter NiCl_2_ doped phantom bottles. (b) A torso coil is centered on top of the polyurethane foam phantom. (c) The uniformity linearity phantom is placed on top of the coil to provide a lossy signal source. (d) Coronal T_1_‐weighted gradient echo MRIs of the phantom (TE/TR: 3.4/147 ms, 60^0^, 2 × 2 × 5 mm, 300 Hz/pixel, 147 s) were acquired for each of the six coil elements using a 45‐cm field of view. The second element (image inside dashed lines) has low SNR indicating failure. MRI, magnetic resonance imaging; SNR, signal‐to‐noise ratio.

Common sources of past MRI subsystem failures are summarized in Table 2 from both systems covering a period of 1 year that included combined 2533 treatment fractions in 315 patients. Examples of image quality associated with coil failures are shown in Figs. 14, 15.

**Table 2:** Common sources that impact the reliability of the MRI subsystem.

Source	Mechanism	Manifestation	Frequency per system	Typical return to service time
Torso RF Coils	Broken components	Signal loss, image noise or artifact	6/yr	<30 min†
MRI RTC disconnects	Communications issues	Loss of communications halts operations	<2/month	<30 min
MRI‐GO	Real time feedback	Software disconnects between subsystems	<3/month	<30 min*
Patient table	Sensor failure	Table error halts operations	<1/month	SW: <30 minHW: <1.5 days
MLC failure	Stuck MLC	MLC error halts operations	<2/month	<30 min
Gantry	Sensor failure	Rotation error halts operations	<3/yr	SW: 20 minHW: <1.5 days
^60^Co	Delivery errors	Software and source errors halt operations	<2/month	<45 min
Linac	Delivery errors	Software errors halt operations	<6/month	<45 min

MLC: Multi‐leaf collimator, RTC: Radiation therapy control. Return to service times depend on software version and assume a field service engineer is present on site. SW: software repair (e.g., system reboot). HW: hardware repair (e.g., part replacement).

* Return to service times have decreased since April 2019 software patch installation.

† Assumes spare RF coil is available on site, otherwise ~1 day.

**Figure 16:**
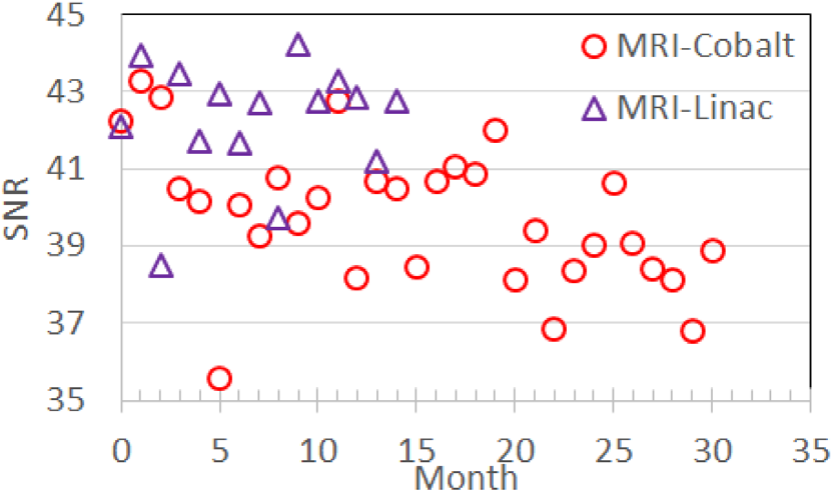
Torso coil SNR measured monthly using the ACR phantom and T_1_ weighted MRIs (TE/TR: 20/500 ms, 90^0^, 1 × 1 × 5 mm, 78 Hz/pixel, 260 s). ACR; American College of Radiology; MRI, magnetic resonance imaging; SNR, signal‐to‐noise ratio.

The monthly stability of the SNRs of the composite torso phased array coils for the MRI‐^60^Co and MRI‐Linac are shown in Fig. 16. The mean (SD) SNR was 39.79 (1.83) for the MRI‐^60^Co and 42.26 (1.53) for the MRI‐Linac.

**Figure 17:**
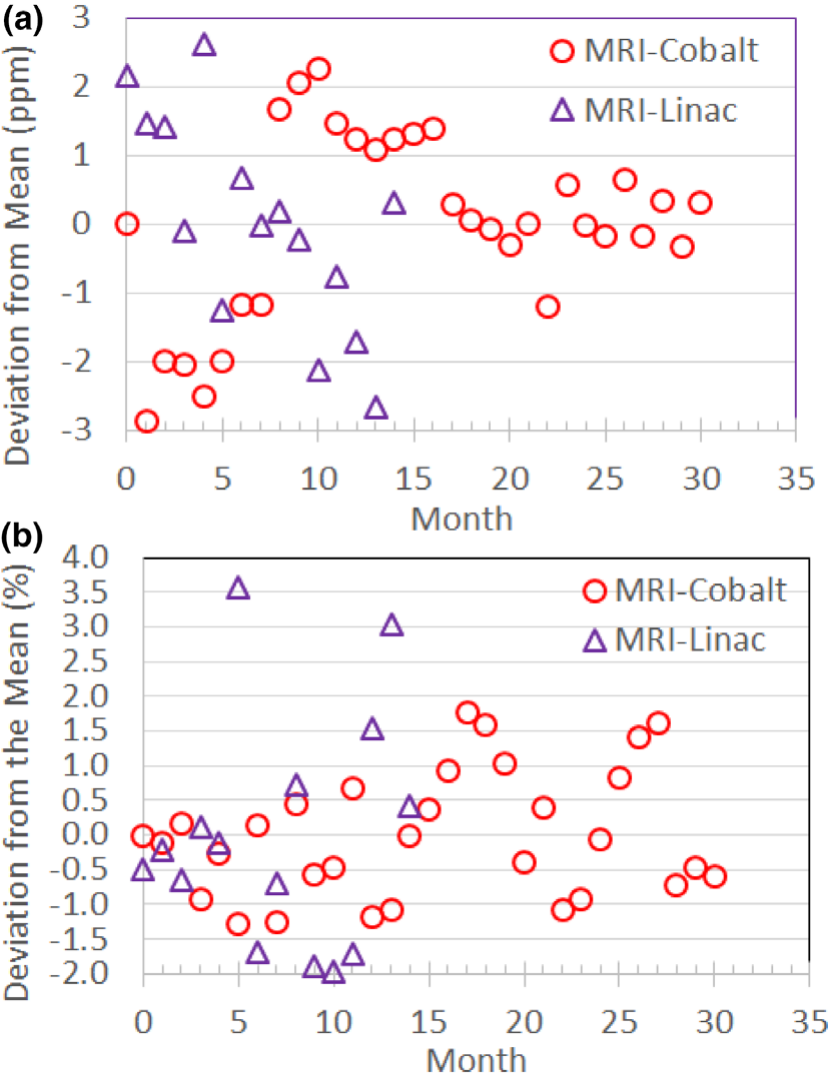
(a) Monthly stability of the Larmor frequency measured as the deviation from the mean. (b) Monthly stability of transmitter reference amplitude measured as the percent deviation from the mean. The MRI‐^60^Co magnet was reramped in Months 1 and 22. The MRI‐Linac experienced high RF noise in Month 5. MRI, magnetic resonance imaging.

The stability of the Larmor frequency and transmitter gain (as represented by the transmitter reference amplitude) for the MRI‐^60^Co and MRI‐Linac are shown in Fig. 17. The standard deviation of the deviation from the mean for the Larmor frequency was 1.41 ppm for the MRI‐^60^Co and 1.54 ppm for the MRI‐Linac. The standard deviation of the deviation from the mean for the transmitter reference amplitude was 0.90% for the MRI‐^60^Co and 1.68% for the MRI‐Linac.

**Figure 18:**
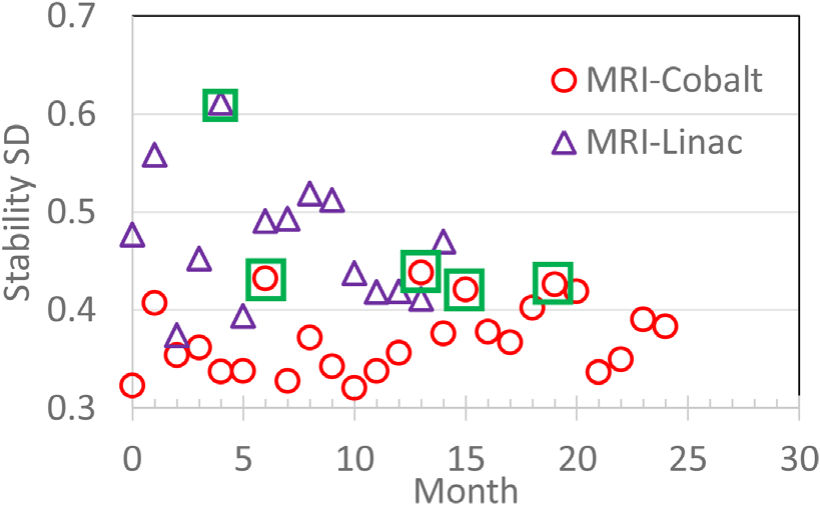
2D TrueFISP monthly image stability standard deviation (SD) results for the MRI‐^60^Co (circles) and MRI‐Linac (triangles). Data that are enclosed by a box corresponded to reports of spike noise during that month. MRI, magnetic resonance imaging.

The monthly standard deviations of the image stability ROIs for the MRI‐^60^Co and MRI‐Linac are shown in Fig. 18. High values of standard deviation (>0.4 for the MRI‐^60^Co and >0.6 for the MRI‐Linac) corresponded to reports of spike noise during that period.

## 4 DISCUSSION

QC programs require a tradeoff between awareness of machine performance and status, and time and effort. Currently, we perform daily, monthly, and annual QC tests for the MRI subsystems of our MR‐IGRTs. Daily QC tests are performed by the radiation therapist while physicists perform the monthly and annual tests. Daily QC tests use a phantom provided by ViewRay, and include table position and geometric accuracy measurements, and image quality and artifact assessments. ACR MRI QC programs stipulate that these tests be performed at least weekly along with high‐ and low‐contrast, center (Larmor) frequency, and transmitter gain measurements. The ACR guidelines do not require monthly tests.

For diagnostic MRIs, daily and weekly QC procedures are typically performed by the MRI technologist. However, ACR QC tests on the low‐field MR‐IGRTs require that the systems be operated as a stand‐alone MRI subsystem that typically requires the presence of the physicist and typically requires >20 min to reboot the system to ensure it is properly operating with the radiation therapy delivery subsystem (RTDS). Obtaining the center frequency and transmitter gain (reference amplitude) on the low‐field MR‐IGRTs requires special procedures to export images or k‐space data files from the MRI subsystem. Automatic daily and weekly MRI QC procedures that can be operated from the RTDS and are supplied by the vendor would greatly benefit the QC process and minimize the impact to the clinical workflow.

### 4.1 Image Noise and Artifact

EMI is a key consideration for the MR‐IGRT since the system combines a source of EMI (the radiation therapy subsystem) with an MRI that is highly vulnerable to EMI. The Linac poses a larger threat than the ^60^Co heads to the quality of the MRI since the Linac uses a high‐voltage linear accelerator and radiofrequency source to accelerate electrons. In turn, the Linac components and the electron beam are vulnerable to the magnetic fields generated by the MRI. The MRI‐Linac employs both magnetic and RF shields to minimize the interaction between the Linac and MRI.

Past sources of radiofrequency interference (RFI) discovered inside the magnet room included a patient camera and a switching DC power supply for the Primalert 10 radiation monitor (Fluke Biomedical, Solon, OH). Sources of RF noise in the gantry cabling were easier to detect using the body coil because of the higher flux between the RF source and the body coil surface area. Use of phased array coils may be less sensitive to RFI since the sensitivity depends on the orientation of the coil surfaces to the noise source. The MRI subsystem passed the NEMA SNR test specification (≥12 with body coil) despite the conspicuous RFI in the MRIs.^14^ Therefore, it is critical to identify and resolve EMI sources before accepting the system based on the vendor's specifications.

During commissioning of the MRI‐Linac, EMI issues were resolved by repairing or replacing cables that had inadequate shielding. Power cables were segregated from signal cables in the gantry and patient table as much as practicable. The offending data filters were disabled. The magnetron tuning rod motor control software was modified to minimize pulsing during MRI acquisition. Nominally, the power to the patient table is automatically turned off to minimize RFI during MRI acquisition. In the MRI‐only mode, the operator must manually disable the power to the patient table to avoid image artifacts.

Metal moving around the MRI can induce quasi‐static EMI. In the case of the MRI‐^60^Co, a pneumatic system is used to drive the sources open and closed. The concussion from the sources opening and closing perturbs the MRI's B_0_. In both MR‐IGRT models, the motion of the multi‐leaf collimators (MLCs) can cause EMI both from eddy currents and motor noise.^15^

For the MRI‐Linac, the large volume of steel shielding in the gantry produced significant dephasing artifacts when the gantry was in motion.^16^ The vendor currently pauses the real‐time display of the cines during gantry rotation although the images can be observed from the MRI subsystem. However, resolution of the dephasing artifacts is desirable because there are several applications that can be applied to the real‐time cines that can benefit treatment including visual respiratory feedback and motion prediction. Dynamic shimming and eddy current methods are now available on commercial MRIs that may be adapted to minimizing gantry motion‐related artifacts.^17^, ^18^

The image artifacts associated with long‐term averaging on the MRI‐Linac indicated that there is a short‐term B_0_ instability and its severity depends on the gantry angle. The choice of long‐term vs short‐term averaging should be based on the timescale of the source of artifact. Long‐term averaging was previously used to minimize image artifacts related to physiological motion that is considered slow relative to the image acquisition time.^12^, ^19^ On the MRI‐^60^Co, long‐term averaging is used to address temporal B_0_ field instabilities associated with the Cobalt heads firing during MRI acquisition.

In the MRI‐Linac, ferrous steel is subject to both hysteresis and eddy currents. Eddy current time constants associated with the 2D cine acquisitions are constrained by the gradient pulse durations (typically ≤1 ms) and are fast compared to the image acquisition time. Eddy currents may induce temperature changes in the steel that alter its magnetization over a longer timescale and affect the stability of the image signal.^20^, ^21^ The TrueFISP signal is a superposition of echo signals that evolved from multiple pathways from earlier RF excitations. Thus, TrueFISP is very sensitive to B_0_ instabilities and inhomogeneities during the acquisition. The short‐term averaging for the ViewRay 2D TrueFISP sequence uses RF phase cycling that can mitigate null band artifacts whereas long‐term averaging does not.^22^

### 4.2 Field Homogeneity and linearity

The main disadvantages of MR‐IGRT vs x ray based IGRT are the spatial distortions that occur primarily due to gradient nonlinearities, and secondarily due to magnetic field inhomogeneities. Distortion correction, high receiver bandwidths, and use of spin echo sequences can mitigate these distortions particularly for 3D acquisitions. However, 2D selective excitations will be affected by distortions if the slice with the tracking target is located far away from isocenter. Fortunately, the Viewray patient setup is typically based on the 3D acquisitions while the 2D acquisitions are primarily used for tracking and beam gating. As with diagnostic MRIs, it is critical to position the target as close to isocenter as possible to minimize geometric distortion. Unfortunately, patient‐specific immobilization devices and arms‐up treatment positions often necessitate that the target be significantly off‐center in the 70‐cm wide bore of the current MR‐IGRT systems.

Based on the spherical phantom measurements, the field inhomogeneities for the MRI‐^60^Co are an order of magnitude smaller than typical pixel bandwidths for the default 2D (>1 kHz/pixel) and 3D (>530 Hz/pixel) TrueFISP acquisitions. However, T_1_‐ and T_2_‐ weighted sequences with pixel bandwidths <100 Hz/pixel were recently FDA‐approved for the 0.35 T MRI‐Linac. Hence, minimization of field inhomogeneities is important to minimize geometric distortions.

MRI‐GO was designed to address the field homogeneity challenges of the MRI‐Linac during 2D cine acquisitions and the data indicates significant improvements in field homogeneity (Fig. 7). The disadvantage of MRI‐GO was the frequent software disconnects related to the real‐time transmission and processing of the gantry angle and shim currents. A recent software update for MRI‐GO has reduced the impact of the software disconnects. MRI‐GO and tune‐up calibrations should also be verified or updated when there are changes to the system that can affect shimming (e.g., gradient driver replacement or recalibration, and main field ramp or shimming).

Unlike the MRI‐^60^CO, the MRI‐Linac also shims the magnet for each patient using the standard shim mode prior to the 3D MRI acquisition used in treatment planning to minimize geometric distortion. The current disadvantage of the standard shim mode is the additional time (~20 s) vs tune‐up shim mode. In addition, the standard shim mode may not work well for all gantry angles especially in regions of high susceptibility like the thorax [Figs. 10(c) and 10(d)]. We recently started homing the MRI‐Linac's gantry angle to 0^0^ prior to each patient session to minimize the effects of field inhomogeneities and eddy currents on image quality, geometric fidelity, and isocenter shifts. Homing the gantry adds <3 min to the clinical workflow. Ideally, the MRI‐Linac should automatically reset the gantry position to 0^0^ at the start of each patient session to ensure the best field shim.

In principle, MRI QC should be assessed at multiple gantry angles. In practice, this is time consuming. ViewRay recommends MRI QC be performed at the gantry angle of 90^0^ (Head 1) for the MRI‐^60^Co and 0^0^ for the MRI‐Linac. These gantry angles correspond to the best field homogeneity using the tune‐up shim mode (Figs. 6, 7).

Our spatial integrity error means and standard deviations were consistent with reported values. Our experiments indicate that the gantry angle had little effect on spatial integrity (Figs. 12, 13). This was expected since the spatial integrity is primarily dictated by the gradient linearity unless the local B_0_ inhomogeneity is comparable to the pixel bandwidth.^23^ It is important to rerun system tests after a major component is replaced or repaired to verify system performance. In addition, the medical physicists must be aware of the system changes conducted by the service engineers since these changes can also impact system performance. For example, after a failure of spatial integrity tests, we subsequently discovered that the vendor had incorrectly recalibrated the gradients on the MRI‐Linac (Fig. 11).

### 4.3 System reliability and stability

Commercial MRI‐IGRT systems combine two distinct subsystems (MRI and radiation therapy delivery). In the case of the MRIdian systems, the radiation therapy control (RTC) is the master and the MRI is the slave. Communications issues or system faults from either subsystem often cause a software disconnection between the two subsystems that halts operations.

The torso phased array receiver coils are the component that fails the most often for the MRI subsystem. The failures are likely related to the coil's light‐weight construction and the wear‐and‐tear imposed on them by the therapists positioning a patient for treatment. ViewRay's flexible torso and head‐and‐neck coils are made from closed‐cell foam and flexible copper conductors. The coils are designed to minimize photon attenuation (0.5%). The vendor does not provide feedback on the cause of the RF coil failure or the procedures used to resolve the failure. RF coils are typically repaired and placed back into service assuming the vendor can identify the cause of the suspected coil failure.

We developed a coil QC method that checks for bad coil elements using a home‐built phantom. The phased array QC procedure can detect failed coil elements. Unfortunately, failures often occur when the flexible coils are bent (e.g., wrapped around a patient), a condition not tested by our current procedure. The torso receiver coils were used for 96% of our MR‐IGRT treatment fractions. Hence, we do not have adequate data to estimate the reliability of the head‐and‐neck coils.

Stability specifications need to be based on baseline measurements since there are no guidelines. However, variations within two to three standard deviations (σ) are typically used for diagnostic MRIs assuming that the QC measurements are performed in a consistent manner.

Monthly variations in SNR measured using the torso phased array coils reached 2.3 and 2.5 σ for the MRI‐^60^Co and MRI‐Linac, respectively. Variations may be caused by differences between coil sensitivities and measurement setup. Based on their different Larmor frequencies, SNR should be 8% higher on the MRI‐Linac vs the MRI‐^60^Co.^24^ We measured SNR to be 6% higher on the MRI‐Linac.

The Larmor frequency varied by less than +/−3 ppm (≤2 σ) in both models over the long term. According to AAPM Report No. 10, the drift rate for superconducting magnets should be ≤0.25 ppm/day during routine operations.^5^ The vendor does not have a long‐term stability specification but does have a short‐term stability specification of <3 ppm/hr that was met during annual QC measurements.

Monthly variations in the reference amplitude reached 2.0 and 2.1 σ for the MRI‐^60^Co and MRI‐Linac, respectively. There is no specification for the transmitter gain stability. Changes in transmitter gain can be indicative of MRI system problems or changes. The transmitter reference amplitude rose either after the magnet was ramped or during a period of frequent image artifact.

Monthly variations in the image signal stability reached 1.8 and 2.2 σ for the MRI‐^60^Co and MRI‐Linac, respectively. High standard deviation values were correlated with RF spike noise issues.

## 5 CONCLUSIONS

MR‐IGRT units are complex systems that integrate an MRI subsystem with a radiation therapy delivery subsystem. The interaction between the two subsystems presents major technical challenges that can affect the quality and reliability of MR‐IGRT. Most of these technical challenges (image noise and artifact, field inhomogeneities, and reliability) were successfully addressed during commissioning and system upgrades for the 0.35 T MRI‐^60^Co and the MRI‐Linac systems. Some issues like component failures and operational interruptions from subsystem software disconnects require further attention. MRI QC will further benefit from the availability of fast automated daily and weekly measurements that can be easily executed by the radiation therapist.

## CONFLICT OF INTEREST

Dr. Green receives speaking fees from ViewRay. Dr. Mutic has consulted for ViewRay. Washington University in St. Louis has a master research agreement and receives research funding from ViewRay unrelated to this study.
